# Pregnant Women and Endocrine Disruptors: Role of P2X7 Receptor and Mitochondrial Alterations in Placental Cell Disorders

**DOI:** 10.3390/cells11030495

**Published:** 2022-01-31

**Authors:** Sophie Fouyet, Elodie Olivier, Pascale Leproux, Mélody Dutot, Patrice Rat

**Affiliations:** 1 Université de Paris, CNRS CiTCoM, 75006 Paris, France; elodie.olivier@u-paris.fr (E.O.); pascale.leproux@u-paris.fr (P.L.); melody.dutot@yslab.fr (M.D.); patrice.rat@u-paris.fr (P.R.); 2 Laboratoires Léa Nature, Recherche & Développement, 17180 Périgny, France; 3 Yslab, Recherche & Développement, 29000 Quimper, France

**Keywords:** lung toxicity, skin toxicity, placental toxicity, endocrine disruptors, P2X7 receptor, mitochondrial alterations, apoptosis

## Abstract

In pregnant women, the lungs, skin and placenta are exposed daily to endocrine-disrupting chemicals (EDCs). EDCs induce multiple adverse effects, not only on endocrine organs, but also on non-endocrine organs, with the P2X7 cell death receptor being potentially the common key element. Our objective was first to investigate mechanisms of EDCs toxicity in both endocrine and non-endocrine cells through P2X7 receptor activation, and second, to compare the level of activation in lung, skin and placental cells. In addition, apoptosis in placental cells was studied because the placenta is the most exposed organ to EDCs and has essential endocrine functions. A total of nine EDCs were evaluated on three human cell models. We observed that the P2X7 receptor was not activated by EDCs in lung non-endocrine cells but was activated in skin and placenta cells, with the highest activation in placenta cells. P2X7 receptor activation and apoptosis are pathways shared by all tested EDCs in endocrine placental cells. P2X7 receptor activation along with apoptosis induction could be key elements in understanding endocrine placental and skin disorders induced by EDCs.

## 1. Introduction

Endocrine-disrupting chemicals (EDCs) are defined by the World Health Organization as exogenous substances or mixtures that alter function(s) of the endocrine system and consequently cause adverse health effects in an intact organism, or its progeny, or (sub)populations [1]. EDCs are mostly found in personal care products, food contaminants, metals, additives and plastics and even some medications. Pregnant women and children are the most vulnerable populations to be affected by EDCs exposure, and the effects of exposure to EDCs may not become evident until later in life. Adverse pregnancy outcomes induced by EDCs can be harmful for both the mother and the baby.

Chronic exposure to EDCs can occur through breathed air, food and daily life products such as cosmetics; therefore, inhalation, ingestion and skin contact are the main routes of exposure. In pregnant women, the lungs, skin and placenta (through blood circulation) are then continuously exposed to EDCs that have been reported to induce multiple adverse effects including asthma [2], urticaria, allergic contact dermatitis and skin aging [3], preterm birth and the worst case scenario of preeclampsia [4,5,6,7,8,9,10]. It is then obvious that EDCs act not only in endocrine organs but also in non-endocrine organs, and exert pleiotropic effects.

Whether EDCs share a common mechanism of action on both endocrine and non-endocrine organs remains unclear. The above-cited pathologies that EDCs can induce are different in terms of clinical features, morbidity and consequences for health, but we observed in the literature that the P2X7 receptor seems to be implicated in their development [11,12,13,14,15,16]. P2X7 receptor activation is reported to be involved in multiple pathologies from immune disorders to degenerative diseases [17,18,19,20]. The P2X7 receptor is a ubiquitous membrane receptor that induces many intracellular signaling pathways after alterations of the ion permeability or after formation of a large pore, depending on the duration of the stimulus. Pore formation after prolonged activation of the P2X7 receptor leads to apoptosis via multiple mechanisms including caspase-8 and caspase-9 activation, ROS production, mitochondrial dysfunction and caspase-3/7 activation [21,22,23,24]. In human placental cells, we previously showed that P2X7 receptor activation plays a pivotal role in toxicity induced by both known EDCs such as bisphenols (bisphenol A, bisphenol F and bisphenol S) [25] and suspected EDCs such as benzo[a]pyrene [26].

**Figure 1:**
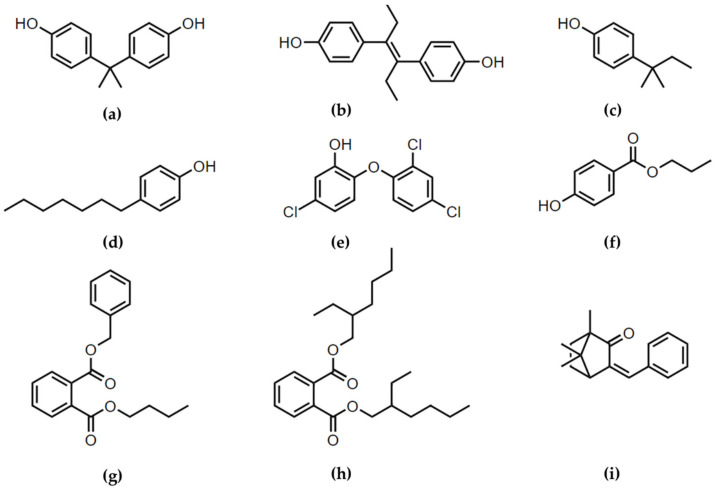
EDCs chemical structures: (**a**) bisphenol A, (**b**) diethylstilbestrol, (**c**) 4-tert-amylphenol, (**d**) 4-heptylphenol, (**e**) triclosan, (**f**) propylparaben, (**g**) benzyl butyl phthalate, (**h**) DEHP and (**i**) 3-benzylidene camphor.

The question that we raised is then: do EDCs share P2X7 receptor activation as a common cellular mechanism of toxicity in pregnant women organs? Reported alterations of the placenta upon EDCs exposure (preterm birth, preeclampsia) are more dangerous for both the mother and her fetus than reported alterations of the lungs and skin (asthma and dermatitis). Another question that can therefore be asked is: should the level of P2X7 receptor activation after EDCs exposure be the same in the lungs, skin and placenta? Lungs being a non-endocrine organ, skin being closely related to the endocrine system and referred to as steroidogenic tissue [27] and placenta being an endocrine organ, we hypothesize that the level of P2X7 receptor activation induced by EDCs can be classified as follows: higher in placenta than in skin and higher in skin than in lungs. To provide preliminary in vitro answers, we studied P2X7 receptor activation after incubation with EDCs in human cells that express P2X7 receptor: human lung A549 cells [28], human keratinocytes HaCaT cells [29] and human placental JEG-Tox cells [25]. A total of nine EDCs belonging to different chemical families were selected for their susceptibility to be either inhaled, directly applied to the skin and/or ingested (Figure 1). In each case, EDCs can pass into the blood circulation after diffusion through skin and pulmonary barriers and digestion, ultimately reaching the placenta where they can accumulate [30]. The placenta being the most exposed organ, further investigations were performed to study apoptosis through the measurements of caspases-3, -8 and -9 activity, mitochondrial potential and chromatin condensation. The EDCs we tested in lung cells were selected because of their pulmonary exposition route: bisphenol A and benzyl butyl phthalate are plasticizers which can be contained in air or dust. The same rationale was used in skin cells: propylparaben is a commonly used preservative in cosmetics and 3-benzylidene camphor serves as an ultraviolet (UV) filter in sunscreen products. The EDCs tested in placental cells were selected because they are the most abundant EDCs in pregnant women fluids and placentas [8,9,31,32,33]: bisphenol A, 4-heptylphenol (additive found in lubricants and greases), 4-tert-amylphenol (germicide and fumigant), phthalates (benzyl butyl phthalate and di(2-ethylhexyl) phthalate, DEHP), propylparaben, 3-benzylidene camphor and triclosan (biocide used in cosmetics). Diethylstilbestrol, a well-known EDC, was also tested because it was prescribed to pregnant women between 1940 and 1970 to prevent miscarriage, premature labor and related complications of pregnancy. For the record, the EDCs detected in placentas are listed because of their endocrine properties either as substances of very high concern (SVHC) under REACH legislation, or restricted to people aged under 3 years in cosmetic products by the European Commission (regulation 358/2014), or banned by the U.S. Food and Drug Administration.

## 2. Materials and Methods

Chemicals and reagents: Minimum essential Medium (MEM), Roswell Park Memorial Institute (RPMI) 1640 medium, Fœtal Bovine Serum (FBS), 2 mM glutamine, 100 U/mL penicillin and 100 µg/mL streptomycin, trypsin-EDTA 0.05% and Phosphate Buffer Saline (PBS) were provided by Gibco (Paisley, UK) and cell culture plastics such as flasks and microplates by Corning (Schiphol-Rijk, The Netherlands). Cell Event^TM^ Caspase-3/7 green ReadyProbesTM, YO-PRO-1^®^, JC-1 and Hoechst 33342 probes were obtained from Thermo Fisher Scientific (Waltham, Massachusetts, USA) and Caspase-Glo^®^ 8 Assay and Caspase-Glo^®^ 9 Assay from Promega (Madison, WI, USA).

All chemicals were purchased from Sigma-Aldrich (Saint Quentin Fallavier, France). Di(2-ethylhexyl)phthalate was dissolved in culture medium. Benzyl butyl phthalate and propylparaben were dissolved in absolute ethanol. Bisphenol A, diethylstilbestrol, 4-tert-amylphenol, 4-heptylphenol, triclosan and 3-benzylidene camphor were dissolved in dimethylsulfoxyde (DMSO). Stock solutions were stored at –20 °C and work solutions were obtained after a 1/1 000 dilution in culture medium. The final concentration of absolute ethanol and DMSO on cells was less than or equal to 0.1%.

Cells culture: Human placental JEG-3 cell line (ATCC HTB-36), human lung A549 cell line (ATCC CCL-185) and human keratinocytes HaCaT cell line (Cell lines service-CLS-Germany) were cultured under standard conditions (37 °C, saturated humidity and 5% CO_2_), in Minimum Essential Medium (MEM) for JEG-3 cells and Dulbecco’s Modified Eagle Medium (DMEM) for HaCaT and A549 cells, supplemented with 10% FBS, 1% L-glutamine, 0.5% penicillin and streptomycin in 75 cm^2^ polystyrene flasks. Confluent cells were detached by trypsin-EDTA incubation. Cells were seeded into 96-well culture microplates at a density of 80,000 cells/mL (200µL/wells for JEG-3 and HaCaT, 100µL/wells for A549) for analysis. Cultures were kept at 37 °C for 24 h.

Cell incubation: To study the P2X7 receptor and its relationship between apoptosis, the cells were preincubated with either PBS or P2X7 antagonist Brilliant Blue G (BBG) at 25 µM for 15 min [34]. After removal of PBS and BBG, the cells were incubated for 72 h with bisphenol A (5, 10 and 20 µM), diethylstilbestrol (3.75, 7.5 and 15 µM), 4-tert-amylphenol (1, 10 and 50 µM), 4-heptylphenol (1, 10 and 50 µM), triclosan (0.1, 1 and 10 µM), propylparaben (20, 50 and 100 µM), benzyl butyl phthalate (1, 10 and 50 µM), DEHP (1, 10 and 50 µM) and 3-benzylidene camphor (1, 10 and 50 µM) in MEM with 2.5% FBS according to Olivier et al.’s protocol that describes the JEG-Tox model [35] or DMEM with 2.5% FBS for the HaCaT and A549. Concentrations tested in placental cells were selected according to the literature and the same concentrations were used to study the lung and skin cells [6,7,9,32,36,37].

Cell viability: Neutral Red assay. The Neutral Red solution at 0.4% (m/v in water) was diluted in cell culture medium to obtain a working concentration of 50 µg/mL. Neutral Red working solution was distributed in the plates for a 3 h incubation time at 37 °C. The cells were then rinsed with PBS and lysed with a solution of ethanol–water–acetic acid (50.6/48.4/1, *v*/*v*/*v*). After homogenization, the fluorescence signal was scanned (λ_ex_ = 540 nm, λ_em_ = 600 nm) using a Spark^®^ microplate reader (Tecan, Männedorf, Switzerland).

Cell death P2X7 receptor activation: YO-PRO-1^®^ assay. P2X7 cell death receptor activation was evaluated using the YO-PRO-1^®^ assay [38]. The YO-PRO-1^®^ probe only enters into cells after pore opening induced by P2X7 receptor activation and binds to DNA, emitting fluorescence. A 1 mM YO-PRO-1 stock solution was diluted at 1/500 in PBS just before being used and distributed in the wells of the microplate. After a 10 min incubation time at room temperature, the fluorescence signal was read (λ_ex_ = 485 nm, λ_em_ = 531 nm) using the Spark^®^ microplate reader.

Caspase-8, -9 activity: Caspase-Glo^®^ Assays. Caspase-8 and -9 activities were evaluated using the Caspase-Glo^®^ 8 and 9 assay kits, respectively. The assay was performed according to the manufacturer’s instructions. Luminescence was quantified using a Spark^®^ microplate reader.

Caspase 3 activity: CellEvent^TM^ Caspase-3/7 Green Detection Reagent. Caspase-3 activity was evaluated using the CellEvent^TM^ Caspase3/7 Green Detection Reagent. Cell Event^TM^ Caspase-3/7 Green Detection reagent was diluted in PBS with 2.5% FBS to a final concentration of 8µM. The cells were incubated with the reagent for 30 min and then rinsed with PBS. The cells were observed under fluorescence microscopy and pictures were captured under the same acquisition parameters by Evos FL fluorescence microscope (Thermo Fisher Scientific).

Mitochondrial membrane potential: To determine mitochondrial potential we used the membrane potential-sensitive probe JC-1, which forms J-aggregates (with red color) at higher potential and JC-1 monomers (with green color) at low membrane potential, and the ratio between the red and green signals is a measure of mitochondrial potential. The dye at 6.5µg/mL of PBS was added to living adherent cells. The microplate was incubated at 37 °C for 15 min and then read at λ_ex_ = 485 nm and λ_em_ = 600 nm for the red fluorescence and λ_ex_ = 485 nm and λ_em_ = 520 nm for the green fluorescence. Carbonyl cyanide m-chlorophenylhydrazone (CCCP, Sigma-Aldrich) was used as a positive control for mitochondrial depolarization.

Chromatin condensation: Hoechst 33342 assay. Chromatin condensation was evaluated using the Hoechst 33342 assay. The Hoechst 33342 fluorescent probe enters and intercalates into DNA in living and apoptotic cells. The fluorescent signal is proportional to chromatin condensation. A 0.5µg/mL Hoechst 33342 solution was distributed in the wells of the microplate. The fluorescence signal was read after a 30 min incubation time at room temperature (λ_ex_ = 350 nm, λ_em_ = 450 nm) using a Spark^®^ microplate reader.

Results exploitation and statistical analysis: Results are expressed in percentage or fold change compared with control cells and presented as means of at least three independent experiments ± standard errors of the mean. Statistical analysis was performed using Prism software (version 8, GraphPad software, La Jolla, CA, USA). The normal distribution of the data was confirmed by D’Agostino–Pearson test. Then, a one-way analysis of variance for repeated measures followed by a Dunnett’s test with risk α set at 5% was performed to compare EDCs incubation with control (*p*-value expressed as follows: *) and a *t*-test was used to compare results in the presence of BBG with results in its absence (*p*-value expressed as follows: #).

## 3. Results

### 3.1. Cell Viability

We investigated A549, HaCaT and JEG-Tox cells viability after incubation with EDCs, using the neutral red assay. Any concentration inducing a loss of cell viability greater than or equal to 30% was considered as cytotoxic (ISO 10993-5:2009).

**Figure 2:**
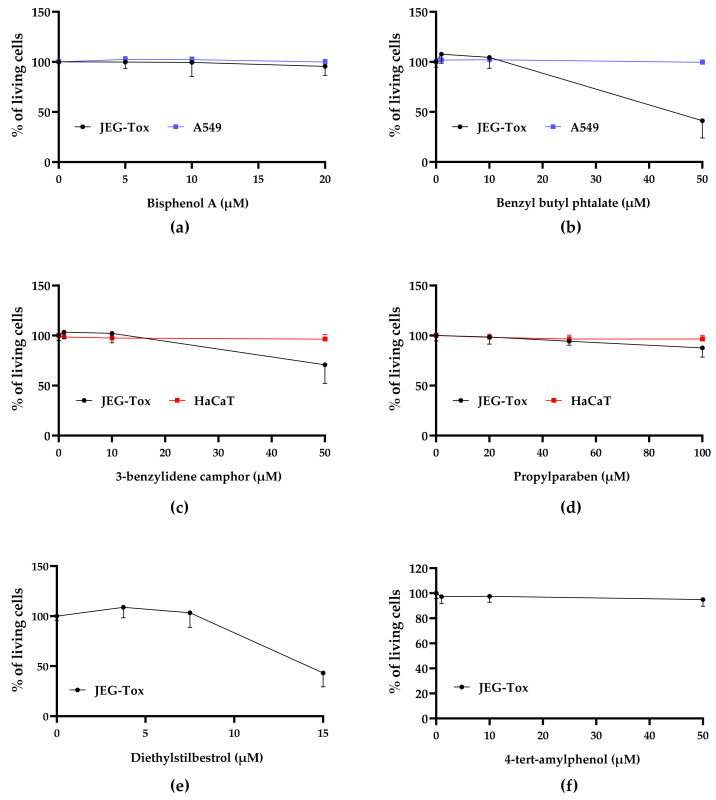

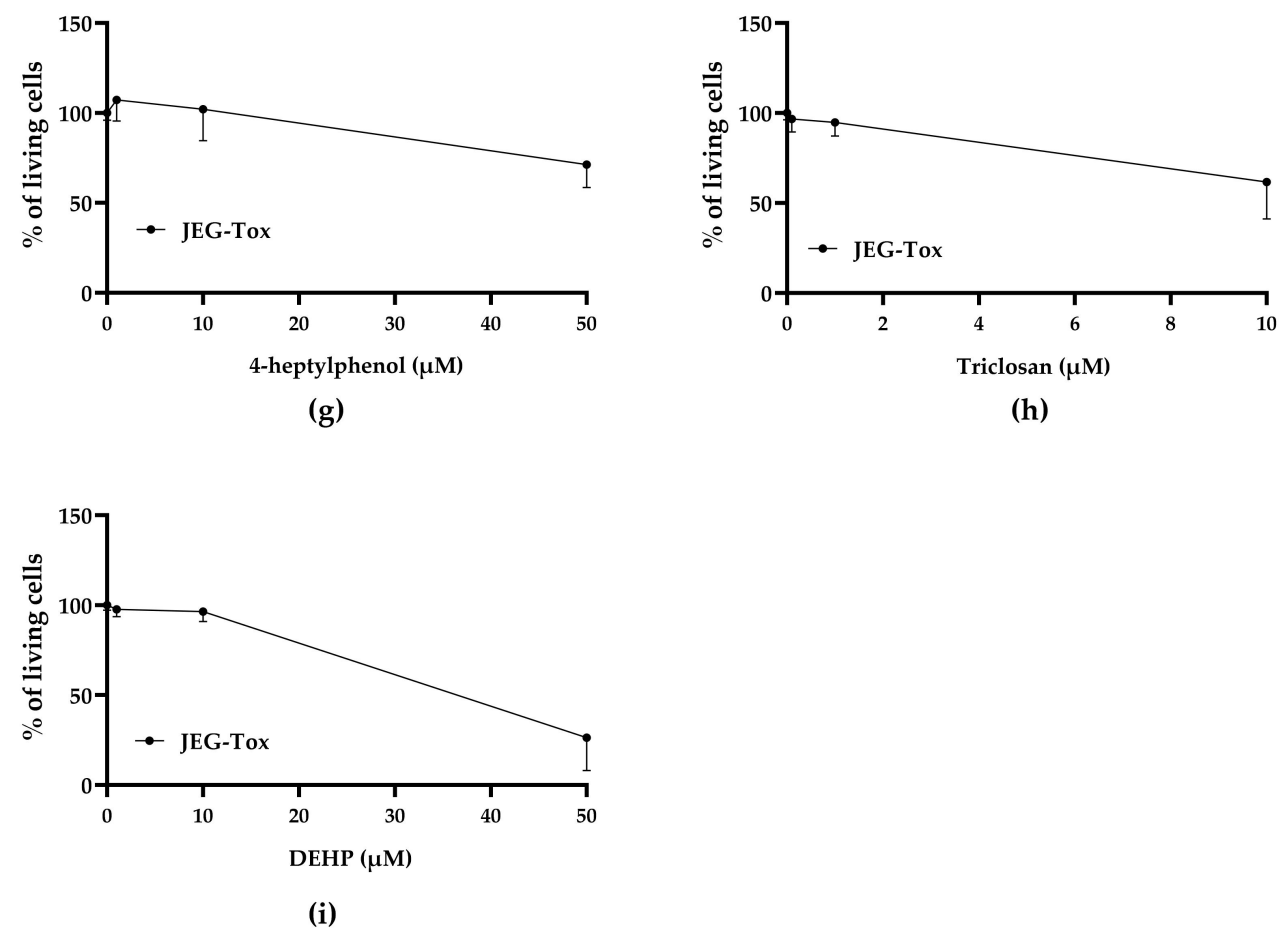
Cell viability was evaluated using the neutral red assay after (**a**) bisphenol A and (**b**) benzyl butyl phthalate incubation for 72 h in A549 cells (blue bars) and JEG-Tox cells (black bars); (**c**) 3-benzylidene camphor and (**d**) propylparaben in HaCaT (red bars) and JEG-Tox cells (black bars); (**e**) diethylstilbestrol, (**f**) 4-ter-amylphenol, (**g**) 4-heptylphenol, (**h**) triclosan and (**i**) DEHP in JEG-Tox cells (black bars).

Bisphenol A and benzyl butyl phthalate, 3-benzylidene camphor and propylparaben had no cytotoxic effects at the tested concentrations in A549 cells (Figure 2a,b) and HaCaT cells (Figure 2c,d), respectively. No loss of JEG-Tox cell viability was observed with neither bisphenol A (Figure 2a), nor with 4-tert-amylphenol (Figure 2f). Propylparaben slightly reduced cell viability at 100µM but remains not cytotoxic (87%, Figure 2d). JEG-Tox cells viability was reduced with benzyl butyl phthalate (40% at 50 µM, Figure 2b), 3-benzylidene camphor (70%, Figure 2c), diethylstilbestrol (40% at 15 µM, Figure 2e), 4-heptylphenol (45% at 50 µM, Figure 2g), triclosan (60% at 10 µM, Figure 2h) and DEHP (25%, Figure 2i). Cytotoxic concentrations were excluded from subsequent assays.

### 3.2. P2X7 Receptor Activation

**Figure 3:**
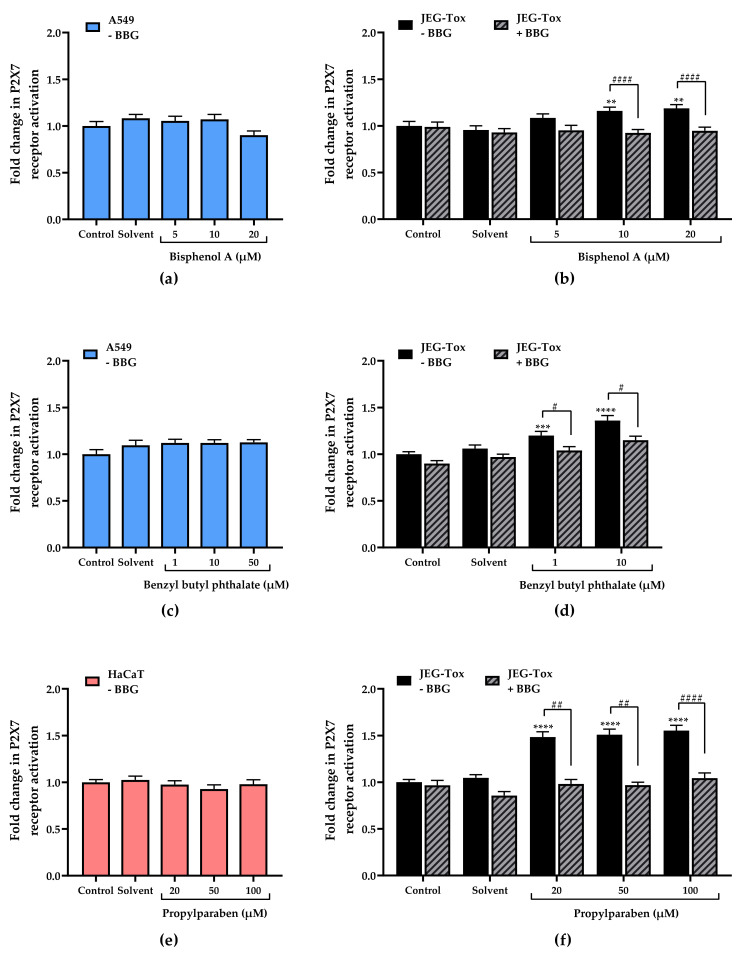

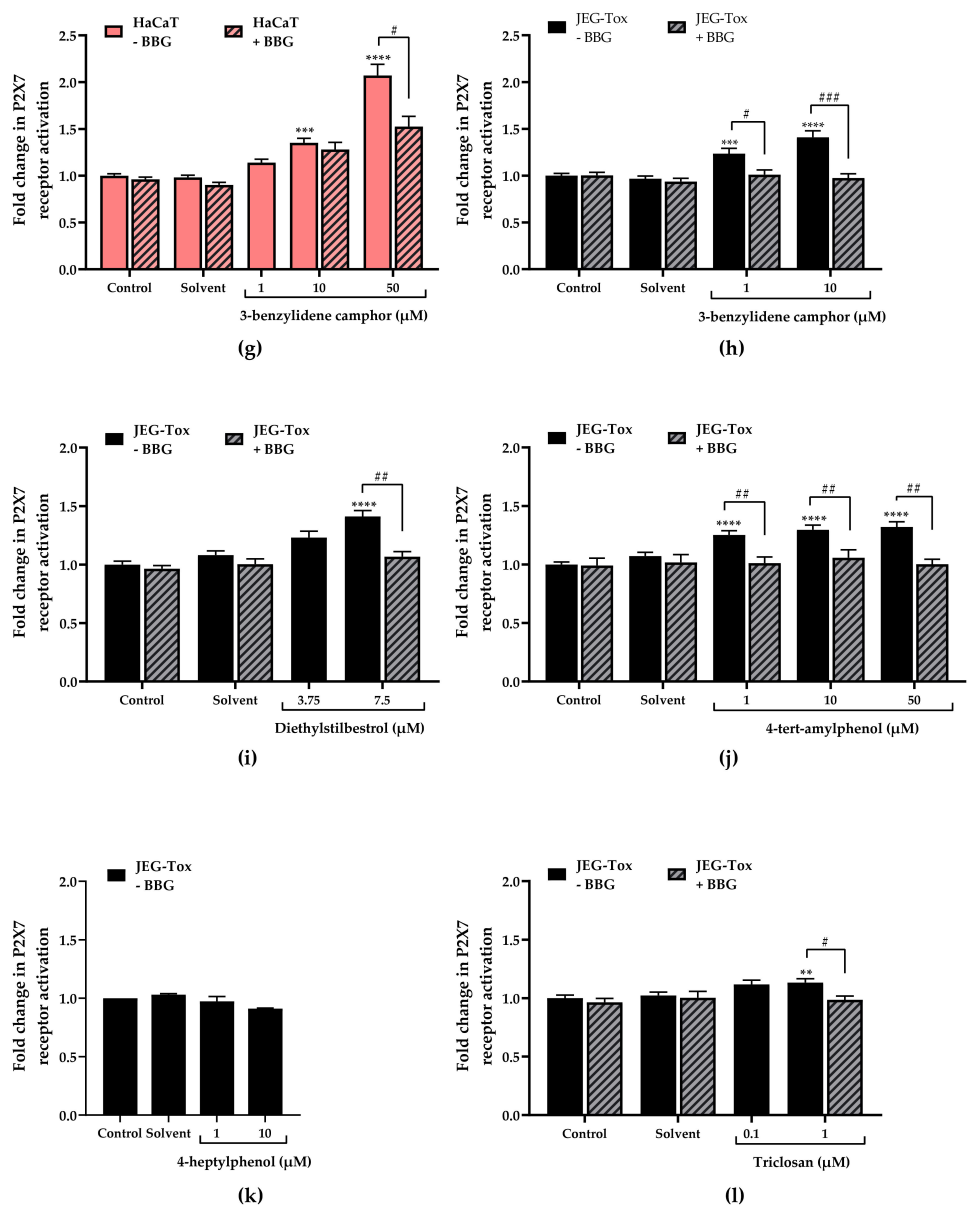

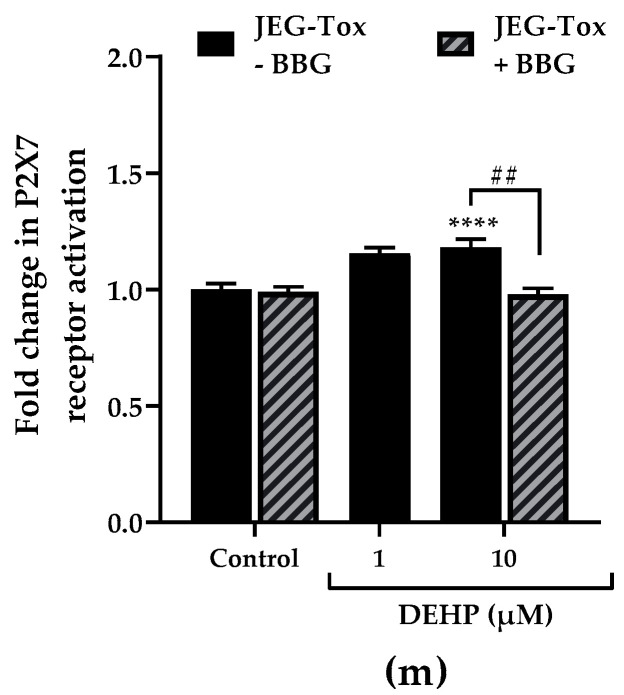
Effects of EDCs on P2X7 receptor activation in A549 cells, HaCaT cells and JEG-Tox cells after incubation for 72 h (YO-PRO-1^®^ assay). The cells were preincubated with either PBS (solid bars) or with Brilliant Blue G at 25 µM (hatched bars) for 15 min. The solutions were removed and A549 cells were incubated with (**a**) bisphenol A and (**c**) benzyl butyl phthalate; HaCaT cells were incubated with (**e**) propylparaben and (**g**) 3-benzylidene camphor; JEG-Tox cells were incubated with (**b**) bisphenol A, (**d**) benzyl butyl phthalate, (**f**) propylparaben, (**h**) 3-benzylidene camphor, (**i**) diethylstilbestrol, (**j**) 4-tert-amylphenol, (**k**) 4-heptylphenol, (**l**) triclosan and (**m**) DEHP. The significance thresholds were **** *p* < 0.0001, *** *p* < 0.001 and ** *p* < 0.01 compared with the control and #### *p* < 0.0001, ### *p* < 0.001, ## *p* < 0.01 and # *p* < 0.1.

P2X7 pore opening, reflecting P2X7 receptor activation, was assessed using the fluorescent YO-PRO-1^®^ assay. There is no effect of bisphenol A and benzyl butyl phthalate on P2X7 receptor in A549 cells (Figure 3a,c).

In HaCaT cells, 3 benzylidene camphor induced a high fold change in P2X7 receptor activation (×1.35 at 10 µM and ×2.07 at 50 µM compared with the control, Figure 3g). The activation induced at 50µM was significantly inhibited by the P2X7 receptor antagonist BBG. Propylparaben had no effect on P2X7 receptor in HaCaT cells (Figure 3e).

In JEG-Tox cells, 4-heptylphenol had no effect on P2X7 receptor activation (Figure 3k). BPA, DEHP and triclosan were the substances that induced the slightest fold changes in P2X7 receptor activation (×1.16 at 10 µM and 1.18 at 20 µM in Figure 3b; ×1.18 at 10 µM in Figure 3l and ×1.13 at 1 µM in Figure 3m, respectively compared with the control). Diethylstilbestrol, 4-tert-amylphenol, butyl benzyl phthalate and 3-benzylidene camphor induced intermediate fold changes (×1.41 at 7.5 µM in Figure 3i; ×1.25 at 1 µM, ×1.30 at 10 µM, ×1.32 at 50 µM in Figure 3j; ×1.20 at 1 µM and 1.36 at 10 µM in fig. 3d; ×1.23 at 1 µM and 1.41 at 10 µM, Figure 3h). Propylparaben was the most potent activator (×1.48 at 20 µM, ×1.51 at 50 µM, ×1.55 at 100 µM, Figure 3f). All the P2X7 receptor activations were significantly inhibited by the P2X7 receptor antagonist BBG (grey hatched bars).

### 3.3. EDCs Effects on Apoptosis in JEG-Tox Cells

The P2X7 receptor was activated by all the tested EDCs except one (4-heptylphenol) in JEG-Tox cells. As the P2X7 cell death receptor is known to trigger apoptosis, we studied apoptosis in JEG-Tox cells through the assessment of caspase-8, caspase-9 and caspase-3 activities, mitochondrial membrane potential and chromatin condensation.

#### 3.3.1. Caspase-8, Caspase-9 and Caspase-3 Activity

**Figure 4:**
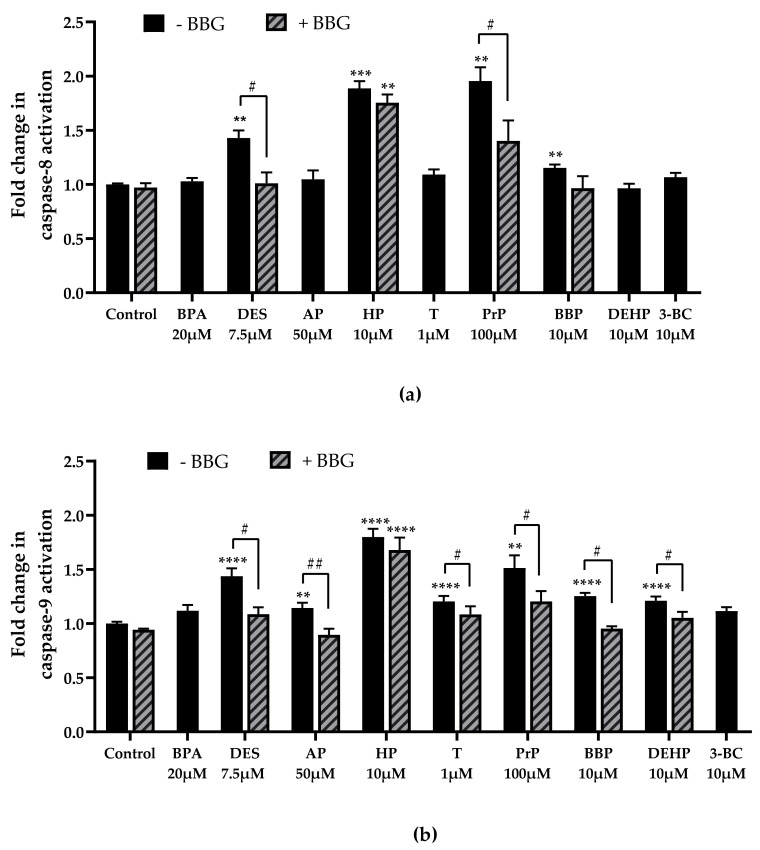
Bisphenol A (BPA), diethylstilbestrol (DES), 4-tert-amylphenol (AP), 4-heptylphenol (HP), triclosan (T), propylparaben (PrP), benzyl butyl phthalate (BBP), DEHP and 3-benzylidene camphor (3BC) effects on caspase-8 (**a**) and caspase-9 (**b**) activity in JEG-Tox cells. The cells were preincubated with either PBS (solid bars) or BBG at 25µM (hatched bars) for 15 min. The solutions were removed and the cells were incubated with EDCs for 72 h. For clarity’s sake, EDCs abbreviations are used in Figure 4, Figure 6 and Figure 7**.** The significance thresholds were **** *p* < 0.0001, *** p < 0.001 and ** *p* < 0.01 compared with the control and ## *p* < 0.01 and # *p* < 0.1.

**Figure 5:**
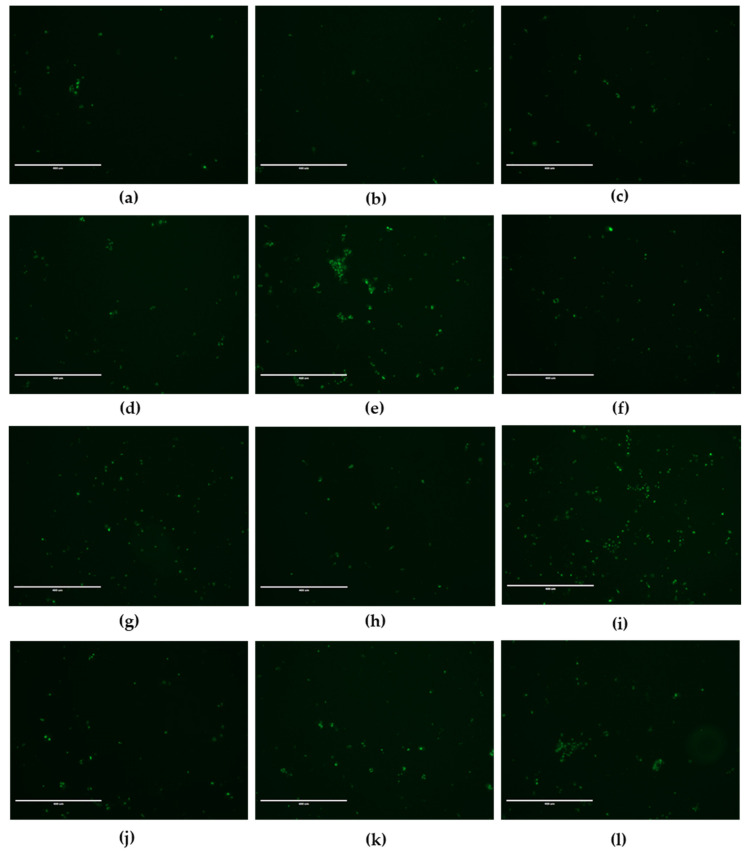
Fluorescence microscopy images of JEG-Tox cells stained for caspase-3/7 activity. After 72 h incubation with (**a**) control, (**b**) DMSO, (**c**) ethanol, (**d**) bisphenol A at 20 µM, (**e**) diethylstilbestrol at 7.5 µM, (**f**) 4-tert-amylphenol at 50 µM, (**g**) 4-heptylphenol at 10 µM, (**h**) triclosan at 1 µM, (**i**) propylparaben at 100 µM, (**j**) benzyl butyl phthalate at 10 µM, (**k**) DEHP at 10 µM and (**l**) 3-benzylidene camphor at 10 µM, the cells were stained using Caspase-3/7 Green ReadyProbes™. Representative images from three independent experiments are shown. Bar scale: 400 µm. Magnification: 10×.

The activity of initiator caspases (8 and 9) and the activity of executioner caspase-3 are reported in Figure 4 and Figure 5.

Diethylstilbestrol, 4-heptylphenol, propylparaben and benzyl butyl phthalate significantly activated caspase-8 (×1.43 at 7.5 µM; ×1.89 at 10 µM; ×1.95 at 100 µM and ×1.15 at 10 µM, respectively, Figure 4a). P2X7 antagonist BBG significantly inhibited caspase-8 activity induced by diethylstilbestrol (×1.43 without BBG and ×1.01 with BBG, Figure 4a), propylparaben (×1.95 without BBG and ×1.40 with BBG, Figure 4a) and benzyl butyl phthalate (×1.15 without BBG versus x0.96 with BBG, Figure 4a) but had no effects on capase-8 activation induced by 4-heptylphenol. Bisphenol A, 4-tert-amylphenol, triclosan, DEHP and 3-benzylidene camphor had no effect on caspase-8 activity (Figure 4a).

Diethilstilbestrol, 4-tert-amylphenol, triclosan, propylparaben, benzyl butyl phthalate and DEHP triggered caspase-9 activation which was reversed with BBG (×1.43 without BBG versus ×1.01 with BBG at 7.5 µM of diethylstilbestrol; ×1.14 without BBG versus ×0.90 at 50 µM of 4-tert-amylphenol; ×1.20 without BBG versus ×1.08 at 1 µM of triclosan; ×1.51 without BBG versus ×1.20 with BBG at 100 µM of propylparaben; ×1.25 without BBG versus x0.95 with BBG at 10 µM of benzyl butyl phthalate; ×1.21 without BBG versus ×1.05 with BBG at 10 µM of DEHP; respectively, Figure 4b). 4-heptylphenol also significantly activated caspase-9 compared with the control, but this activity was not inhibited by P2X7 antagonist BBG (×1.80 at 10 µM without BBG and ×1.68 with BBG, Figure 4b).

Caspase-3 activity was induced by diethylstilbestrol at 7.5 µM, 4-heptylphenol at 10 µM, propylparaben at 100µM and 3-benzylidene camphor at 10µM compared to control (Figure 5e,g,I,n).

#### 3.3.2. Mitochondrial Membrane Potential

**Figure 6:**
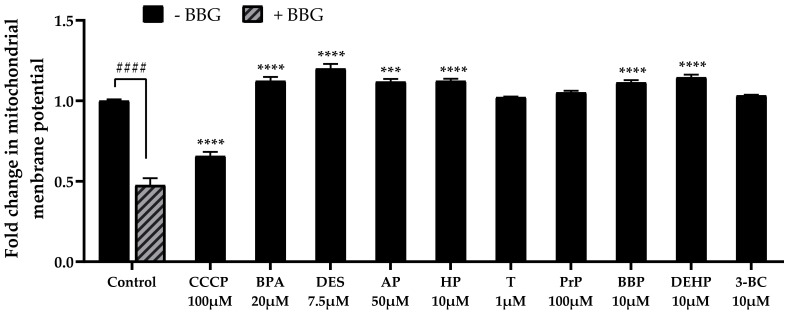
Mitochondrial membrane potential was evaluated using the JC-1 assay after incubation with bisphenol A (BPA), diethylstilbestrol (DES, 4-tert-amylphenol (AP), 4-heptylphenol (HP), triclosan (T), propylparaben (PrP), benzyl butyl phthalate (BBP), DEHP and 3-benzylidene camphor (3BC) for 72 h in JEG-Tox cells. Control cells were preincubated with either PBS (solid bars) or BBG at 25µM (hatched bars) for 15 min. The solutions were removed and the cells were incubated with culture medium (control) and CCCP (positive control) for 15 min. For clarity’s sake, EDCs abbreviations are used in Figure 4, Figure 6 and Figure 7**.** The significance thresholds were **** *p* < 0.0001 and *** *p* < 0.001 compared with the control and #### *p* < 0.0001.

Caspase-9 activation being associated with mitochondrial disruption during apoptosis, we analysed mitochondrial membrane potential with a JC-1 assay. A positive control, CCCP, known to trigger mitochondrial depolarization, was used to ensure that JEG-Tox possessed functional mitochondria. CCCP led to low mitochondrial membrane potential, as expected (x0.65 compared with the control, Figure 6) corresponding to low mitochondrial activity. BBG induced an unexpected weak fluorescence signal in control cells (x0.47 compared with the control without BBG), which could be attributed to an artifact; BBG seems therefore not suitable to study the relationship between mitochondrial membrane potential and the P2X7 receptor.

Elevated mitochondrial membrane potentials were observed after bisphenol A (×1.12 at 20 µM, Figure 6), diethylstilbestrol (×1.20 at 7.5 µM, Figure 6), 4-tert-amylphenol (×1.12 at 50 µM, Figure 6), 4-heptylphenol (×1.12 at 10 µM, Figure 6), benzyl butyl phthalate (×1.11 at 10 µM, Figure 6) and DEHP (x 1.15 at 10 µM, Figure 6), reflecting mitochondrial membrane hyperpolarization. Triclosan, propylparaben and 3-benzylidene camphor had no effect on mitochondrial membrane potential.

#### 3.3.3. Chromatin Condensation

**Figure 7:**
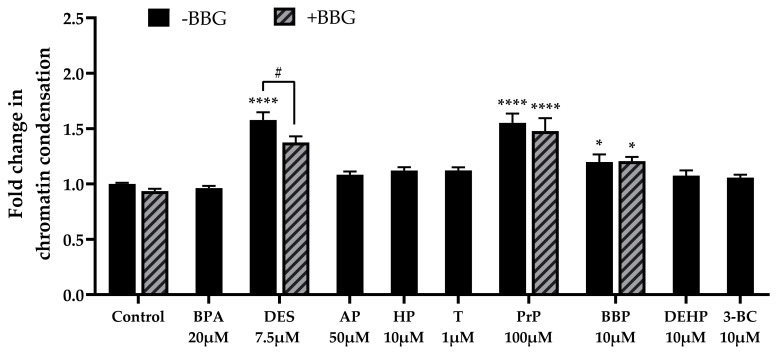
Chromatin condensation was assessed using the Hoechst 33342 assay after bisphenol A (BPA), diethylstilbestrol (DES, 4-tert-amylphenol (AP), 4-heptylphenol (HP), triclosan (T), propylparaben (PrP), benzyl butyl phthalate (BBP), DEHP and 3-benzylidene camphor (3BC) incubation for 72 h in JEG-Tox cells. The cells were preincubated with either PBS (solid bars) or with BBG at 25 µM (hatched bars) for 15 min. The solutions were removed and the cells were incubated with diethylstilbestrol, 4-heptylphenol, butyl benzyl phthalate (BBP) and DEHP. For clarity’s sake, EDCs abbreviations are used in Figure 4, Figure 6 and Figure 7. The significance thresholds were **** *p* < 0.0001 and * *p* < 0.1 compared with the control and # *p* < 0.1.

Apoptosis is characterized by numerous morphological changes, including chromatin condensation that allows DNA fragmentation. We investigated chromatin condensation after incubation with EDCs, using the Hoechst 33342 assay. A statistically significant elevated chromatin condensation was observed with diethylstilbestrol at 7.5 µM (×1.58), propylparaben at 100 µM (×1.55) and benzyl butyl phthalate at 10 µM (×1.20) compared with the control. Chromatin condensation induced by diethylstilbestrol was partially reversed by BBG (×1.58 without BBG versus ×1.37 with BBG, Figure 7). Bisphenol A, 4-tert-amylphenol, 4-heptylphenol, triclosan, DEHP and 3-benzylidene camphor had no significant effect on chromatin condensation.

## 4. Discussion

The objective of the present study was to explore the ability of different EDCs to induce toxicity in different cells by a common cellular mechanism, in particular P2X7 receptor activation. We compared the level of P2X7 receptor activation in human epithelial pulmonary cells, and keratinocytes and placental cells after incubation with EDCs that can be inhaled, directly applied to the skin and/or ingested. The placenta being a crucial organ during pregnancy and the most exposed organ to EDCs, further investigations were performed to study apoptosis, one of the major cell death pathways [39] that can be induced by P2X7 receptor activation [21,40]. Apoptosis is involved in pregnancy disorders [41,42,43].

The three cell lines were selected for their different endocrine properties and because they all express functional P2X7 receptors [13,26,34]. Human epithelial pulmonary A549 cells share similar ultrastructural characteristics and cytochromes expression to in situ type II pneumocytes, the most abundant cells in lungs. Human keratinocyte HaCaT cells have been extensively used to study epidermal homeostasis and its physiopathology. Furthermore, they are metabolically active since they have cytochromes from 1, 2, 3 and 4 families [44]. Human villous trophoblastic placental JEG-3 cells provide an appropriate model to detect placental toxicity [13,25,26]. They are also able to synthesize and secrete hormones.

We selected EDCs reported in the literature to be detected in pregnant women and placentas [8,9,31,32,33]. Exposure to bisphenol A, diethylstilbestrol, 4-tert-amylphenol, 4-heptylphenol, triclosan, propylparaben, benzyl butyl phthalate, DEHP and 3-benzylidene camphor can alter placental functions and therefore induce pregnancy outcomes and complications such as preeclampsia [5,6,7,8,10,45,46]. EDCs were tested at concentrations found in the placenta and we kept the same concentrations to study and compare EDCs effects in lungs and skin, the EDCs pathway’s entry into the body.

The present study is the first to compare EDCs effects on P2X7 receptor activation in three organs present in pregnant woman and possessing different endocrine properties. We highlighted that the P2X7 receptor in lung cells is not sensitive to EDCs and that the P2X7 receptors in skin cells are less sensitive to EDCs than in placental cells where they induce apoptosis.

Bisphenol A and benzyl butyl phthalate did not activate the P2X7 receptor in lung cells, while 3-benzylidene camphor activated the P2X7 receptor in keratinocytes, unlike propylparaben. All of the tested EDCs in human placental cells activated the P2X7 receptor (except 4-heptylphenol). These differences may be explained by the endocrine properties of the different cells. The placenta is considered the most important endocrine organ during pregnancy. Villous trophoblast cells have a lot of steroid or polypeptide hormone receptors and the ability to synthesize, secrete and control different maternal and fetus hormones. Skin, in addition to its main protective function, can also be classified as an endocrine organ endowed with local steroidogenic activities [47,48]. HaCaT keratinocytes possess different steroid receptors [49,50,51] and metabolize progesterone to deoxycorticosterone, cortisone, aldosterone and cortisol [47]. On the contrary, pulmonary epithelial cells do not possess any hormonal receptors and are not able to produce hormones. The lack of P2X7 receptor activation by EDCs in A549 cells did not allow us to arrive at a conclusion on the potential existence of a common mechanism of EDCs in pregnant women organs, but it suggests that P2X7 receptor activation could be a common mechanism in endocrine organs and its activation would be linked to hormonal dysregulation. To confirm this statement, it would be interesting to evaluate P2X7 receptor activation induced by the same EDCs in other endocrine cells, such as adrenal H295 cells, used for the OECD Steroidogenesis Assay (OECD Guidelines for the Testing of Chemicals, Section 4 Test No. 456).

P2X7 receptor activation is known to trigger pregnancy disorders such as preeclampsia and preterm birth [14,16]. In this study, we have shown that EDCs, known in the literature to induce the same disorders, activate the P2X7 receptor. Our results suggest that EDCs could trigger pregnancy disorders through P2X7 receptor activation. P2X7 receptor activation could be a key element in the understanding of placental disorders induced by EDCs.

Prolonged activation of the P2X7 receptor has been linked to apoptosis [21,40]. Depending on the cleaved caspase, apoptosis can be initiated through two major pathways [52]: the extrinsic receptor mediated pathway through caspase-8 activation [53,54] or the intrinsic-mediated pathway, resulting in caspase-9 activation [21,55]. Mitochondrial damages result in cytochrome c release and formation of the apoptosome, a multimeric protein complex containing Apaf-1, cytochrome c and caspase-9, resulting in caspase-9 activation [21,55]. Extrinsic and intrinsic-mediated pathways lead to caspase-3 activation [56], followed by chromatin condensation [57]. Most tested EDCs, despite different chemical structures, triggered apoptosis in placental cells, through different pathways depending on the studied EDC (Table 1). Bisphenol A and 3-benzylidene camphor induced P2X7 receptor activation and mitochondrial membrane potential disturbance. Diethylstilbestrol induced the activation of all the assessed apoptosis markers, meaning that both extrinsic and intrinsic-mediated apoptosis are triggered through P2X7 receptor activation. Propylparaben also induced both extrinsic and intrinsic-mediated apoptosis through P2X7 receptor activation, but contrary to diethylstilbestrol did not induce mitochondrial membrane potential disturbance. Benzyl butyl phthalate induced both extrinsic and intrinsic-mediated apoptosis, but only the intrinsic-mediated apoptosis was associated with P2X7 receptor activation. 4-heptylphenol induced extrinsic and intrinsic-mediated apoptosis, but it was independent from P2X7 receptor activation. 4-tert-amylphenol and DEHP induced P2X7 receptor-dependent intrinsic-mediated apoptosis with mitochondrial membrane potential disturbance. Triclosan induced P2X7 receptor-dependent intrinsic-mediated apoptosis without mitochondrial membrane potential disturbance.

Our study highlights that most of the tested EDCs, belonging to different chemical families, induced mitochondrial alterations through either intrinsic-mediated apoptosis or membrane potential disturbance or both. We were unfortunately unable to link P2X7 receptor activation with mitochondrial membrane potential alteration due to a technical artifact with BBG in the JC-1 assay. The P2X7 receptor and mitochondria have a close connection to ATP. ATP, produced by mitochondria, is the natural ligand of the P2X7 receptor. In syncytiotrophoblast, the majority of ATP is utilized for cholesterol transport and steroidogenesis [58]. Placental mitochondria play a key role in steroidogenesis with huge production of progesterone necessary for maintaining pregnancy [58]. Furthermore, progesterone increases mitochondrial membrane potential [59,60]. As mitochondria are required for steroidogenesis, mitochondrial disorders may contribute to preeclampsia [61,62] and other endocrine diseases such as diabetes mellitus and obesity. All these data suggest that the P2X7 receptor along with mitochondria are specific targets for EDCs to exert their effects on hormones alteration.

## 5. Conclusions

In conclusion, the results of our study suggest that the P2X7 receptor would be a common cellular mechanism of EDCs toxicity in endocrine pregnant women cells. In placental cells, EDCs induce P2X7 receptor activation and mitochondrial alterations, reported to trigger preeclampsia and preterm birth in clinics. P2X7 receptor activation and mitochondrial alterations could be key elements in understanding placental disorders induced by EDCs.

## Figures and Tables

**Table 1:** Summary table of EDCs effects in HaCaT cells, A549 cells and JEG-Tox cell. (/: not tested).

Chemical Family	Chemicals Substances	Cytotoxicity			P2X7 Activation			Caspase-8 Activity	Caspase-9 Activity	Caspase-3 Activity	Mitochondrial Membrane Potential	Chromatin Condensation
		HaCaT Skin Cells	A549 Pulmonary Cells	JEG-Tox Placental Cells	HaCaT Skin Cells	A549 Pulmonary Cells	JEG-Tox Placental Cells	JEG-Tox Placental Cells				
**Bisphenols**	**Bisphenol A**	**/**	**No**	**No**	**/**	**No**	**Yes**	**No**	**No**	**No**	**Yes**	**No**
	**Diethylstilbestrol**	**/**	**/**	**Yes**	**/**	**/**	**Yes**	**Yes**	**Yes**	**Yes**	**Yes**	**Yes**
**Alkylphenols**	**4-tert-amylphenol**	**/**	**/**	**No**	**/**	**/**	**Yes**	**No**	**Yes**	**No**	**Yes**	**No**
	**4-heptylphenol**	**/**	**/**	**Yes**	**/**	**/**	**No**	**Yes**	**Yes**	**Yes**	**Yes**	**No**
**Chlorophenol** **derivatives**	**Triclosan**	**/**	**/**	**Yes**	**/**	**/**	**Yes**	**No**	**Yes**	**No**	**No**	**No**
**Parabens**	**Propylparaben**	**No**	**/**	**No**	**No**	**/**	**Yes**	**Yes**	**Yes**	**Yes**	**No**	**Yes**
**Phthalates**	**Benzyl butyl** **phthalate**	**/**	**No**	**Yes**	**/**	**No**	**Yes**	**Yes**	**Yes**	**No**	**Yes**	**Yes**
	**Di(2-éthylhexyle)** **phthalate** **DEHP**	**/**	**/**	**Yes**	**/**	**/**	**Yes**	**No**	**Yes**	**No**	**Yes**	**No**
**Camphor** **derivatives**	**3-benzylidene** **camphor**	**No**	**/**	**Yes**	**Yes**	**/**	**Yes**	**No**	**No**	**Yes**	**No**	**No**

## Data Availability

The data presented in this study are available on request from the corresponding author.
